# Preschool sleep recommendations are WEIRD

**DOI:** 10.1093/sleep/zsaf025

**Published:** 2025-01-27

**Authors:** Georgie Agar

**Affiliations:** School of Psychology, Aston University, Birmingham, UK; Institute of Health & Neurodevelopment, Aston University, Birmingham, UK

Sleep is critical for healthy development, and yet sleep patterns and requirements are not static throughout the lifespan. In typically developing individuals, sleep parameters gradually change with age so that whilst newborns sleep, on average, for 14.6 hours every 24 hours [1], older adults typically obtain 7 hours of sleep per night [2] with school-age children achieving 8.9 hours [1]. These figures are typically used for guidelines for the recommended amount of sleep children should achieve at each age for optimal functioning. For example, guidance states that preschool children (aged 3-5) should sleep 10-13 hours on a regular basis [3–5]. However, given the significant variability in observed typical sleep patterns, these recommendations may have limited clinical utility when applied to individual children [6–8].

Additionally, there is a lack of population-level sleep data available for many countries [9]. Thus, our understanding of children’s sleep patterns and needs, and therefore intervention targets, is derived predominantly from studies of the Minority World (that is, the United States, Canada, Australia, New Zealand, or Western European countries). In such countries, there is often a focus on autonomy and self-sufficiency, which prioritizes children learning to self-soothe and fall asleep independently. In fact, “good” babies are often praised for being able to sleep through the night alone [10], and parents are criticized for choosing to co-sleep [11]. This leads to the assumption that children should have consolidated sleep from an early age, and the ability to sleep alone, despite arguments that this is neither possible nor optimal for development [12].

Work conducted in the Minority World has influenced our understanding of early sleep patterns and expectations for “normal” sleep in childhood, despite the fact that these countries represent only 7% of young children across the world [13]. Thus, typical sleep recommendations for preschoolers may be described as “WEIRD” (Western, Educated, Industrialized, Rich, and Democratic [14]) and do not take into account differing cultural practices or patterns. It is critical to understand typical sleep patterns from the Majority World in order to manage caregivers’ expectations of child sleep and determine whether sleep interventions are necessary or suitable for a given context.

The paper by Zhang and colleagues [15] in this issue provides the first global evaluation of differences in sleep patterns and practices of preschool children (aged 3-5 years). Although the mean total sleep duration (including naps) did meet the minimum recommendation of 10 hours in all regions [3–5], sleep timing, patterns, and practices differed significantly. Geocultural regions explained up to 30% of the variance in sleep profiles of young children, with the biggest differences in nighttime sleep midpoint and nap duration between regions. In Eastern Europe, Northeast, and Southeast Asia, children had shorter nighttime sleep, poorer sleep efficiency, and longer naps. In South Asia, Latin America, the Middle East, and North Africa, children generally had later sleep schedules with more variability between nights. Children in the Minority World had the least variability in sleep duration between nights.

In addition, Zhang and colleagues [15] highlight different parenting practices associated with sleep. Overall, 22% of parents reported never or occasionally implementing a bedtime routine, and only 18% reported solitary sleeping arrangements for their child. This suggests co-sleeping (and in many cases co-bedding) is the norm for the majority of families across the world, in contrast to current WEIRD expectations. Children in the Minority World were reported to have the most screen time, but children in all regions exceeded the 60 minutes per day recommended by the World Health Organization for this age group [5]. There were geocultural differences in children’s use of electronic devices in the 2 hours prior to bedtime, though these were not examined in relation to sleep parameters.

The authors conclude that children from the Majority World (with the exception of the Pacific Islands) had “less optimal sleep characteristics” than children from the Minority World. This raises the question of what is defined as optimal within different communities, and how this may or may not differ from what actually occurs. Further qualitative work is needed to explore whether caregivers from the Majority World experience these characteristics as problematic.

There is much to commend in this paper, particularly the use of actigraphy to assess children’s typical sleep. This helps to disentangle normative sleep patterns from caregivers’ perceptions of children’s sleep and allows a more precise understanding of which aspects of sleep (i.e. nighttime sleep duration rather than total sleep duration) actually differ between countries. Further research on variation in children’s sleep patterns should strive for similar precision in assessment of sleep parameters. This will allow researchers and practitioners to consider whether an intervention is designed to target total sleep duration (i.e. including daytime napping) or nighttime sleep duration, for example, as these differ substantially between regions. The rigorous methodology is especially impressive given the logistical challenges of collecting data across 37 countries and highlights the need for genuine collaborative research to overcome gaps in the literature relating to global sleep patterns, practices, and expectations.

Whilst the consideration of broader socioecological factors related to parenting and sleep practices is useful, potential associations between screen use, particularly within 2 hours of bedtime, co-sleeping, and sleep characteristics are as yet unexplored. It is not yet clear how identified sleep differences between regions link to children’s health. Further work is needed to determine whether these differences are actually associated with poor health outcomes in the Majority World, and what this may mean for our understanding of “typical” preschool sleep.

In conclusion, the study provides valuable insights into children’s sleep patterns and sleep-related practices from around the globe, including countries where there is very limited prior data. Building on these findings, future work should include geocultural differences in sleep recommendations, which reflect both the location of the child and the expectation of their culture. This is likely to be especially relevant for families from the Majority World living in WEIRD countries, where recommendations for solo sleeping and consistent routines may feel particularly jarring.
